# Plasmodium ARK1 regulates spindle formation during atypical mitosis and forms a divergent chromosomal passenger complex

**DOI:** 10.21203/rs.3.rs-7473364/v1

**Published:** 2025-09-25

**Authors:** Rita Tewari, Annu Nagar, Ryuji Yanase, Mohammad Zeeshan, David J. P. Ferguson, Steven Abel, Sarah Pashley, Akancha Mishra, Anthonius Eze, Edward Rea, Declan Brady, Andrew Bottrill, Sue Vaughan, Karine Le Roch, David Guttery, Anthony Holder, Eelco Tromer, Pushkar Sharma

**Affiliations:** University of Nottingham; National Institute of Immunology; University of Nottingham; University of Nottingham; University of Oxford; University of California Riverside; University of Nottingham; University of Nottingham; University of Glasgow; University of Nottingham; University of Nottingham; University of Warwick; Oxford Brookes University; University of California at Riverside; University of Nottingham; The Francis Crick Institute; University of Groniingen; National Institute of Immunology, New Delhi

**Keywords:** Plasmodium, Aurora kinase, chromosomal passenger complex (CPC), INCENP, spindle, karyokinesis, male gametogony, MTOC, cytokinesis, transmission

## Abstract

Mitosis in *Plasmodium* spp., the causative agent of malaria, is fundamentally different from model eukaryotes, proceeding via a bipartite microtubule organising centre (MTOC) and lacking canonical regulators such as Polo and Bub1 kinases. During schizogony, asynchronous nuclear replication produces a multinucleate schizont, while rapid male gametogony generates an octaploid nucleus before gamete formation. Here, we identify Aurora-related kinase 1 (ARK1) as a key component of inner MTOC and spindle formation, controlling kinetochore dynamics and driving mitotic progression. Conditional ARK1 depletion disrupts spindle biogenesis, kinetochore segregation, karyokinesis and cytokinesis in both stages, and affects parasite transmission. Interactome analysis reveals ARK1 as the catalytic core of a non-canonical chromosomal passenger complex (CPC) containing two divergent inner centromere proteins (INCENPs) but lacking Survivin and Borealin. Comparative genomics indicates this CPC architecture arose early in Apicomplexa, replacing canonical centromere-targeting modules. These findings uncover a distinct mitotic machinery in *Plasmodium* and identify the ARK1–INCENP interface as a potential multistage target for malaria therapeutic intervention.

## Introduction

**Figure 1:**
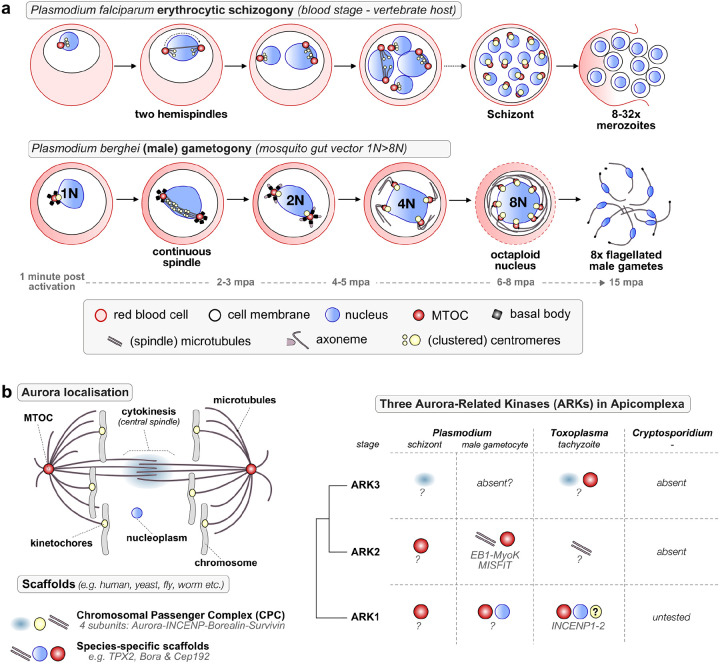
Expansion of the Aurora kinase family among apicomplexan parasites coincides with divergent mitotic mechanisms. **a**. The schematic shows schizogony in the asexual blood stage and male gametogony in *Plasmodium*, highlighting some of the subcellular structures involved (below). **b**. Left: five different cellular locations of Aurora kinases during mitosis/meiosis^10^, based on findings from model organisms. Right: Overview of the location and putative protein complex/interactors of three Aurora kinase paralogs ARK1-2-3 amongst the three apicomplexan parasites *Plasmodium*, *Toxoplasma and Cryptosporidium*.

*Plasmodium* spp., the causative agent of malaria, is a haploid unicellular eukaryote of the phylum Apicomplexa, which is evolutionarily distant from most model organisms and has some unusual divergent features of cell division. During its life cycle, mitosis is closed and typified by two distinct forms: schizogony within vertebrate host cells, and male gametogony within the female mosquito gut (Fig. 1a). During asexual proliferation within host red blood cells (schizogony), repeated asynchronous nuclear division, without chromosome condensation, produces a multinucleate schizont. Subsequent budding produces invasive merozoites to continue the cycle. In contrast, during male gametogony three rounds of rapid genome replication to octaploid (1N to 8N) occur in 8 minutes within the intact nucleus. Karyokinesis and cytokinesis are synchronised with axoneme dynamics by replication and separation of a bipartite microtubule-organising centre (MTOC), chromosome condensation, and complementary de novo basal body formation, leading to the budding of eight flagellated male gametes. These events occur within 12 to 15 min of gametocyte activation, which is probably the most rapid mitosis amongst eukaryotes^1^. Compared to schizogony, mitosis in male gametogony is unusual, with atypical continuous spindles and an MTOC that spans the nuclear envelope connecting the spindle microtubules with those of the axoneme. Male gametes are the only flagellated stage in the parasite’s life cycle, and therefore the only stage where centriole formation and de novo replication of the basal body occurs^1–4^.

Aurora-related kinases (ARKs) are multi-functional mitotic kinases that regulate eukaryotic cell division. They act on spindle pole duplication, kinetochore-microtubule attachment, spindle assembly, mitotic checkpoints and cytokinesis^5–8^. Budding yeast and the amoeba *Dictyostelium discoideum*^9^ have a single ARK to execute all functions, suggesting these to be ancestral to all eukaryotes^7^. However, multiple different lineages (e.g. vertebrates, plants, kinetoplastids) have one or more paralogs that resulted from parallel duplications, which subdivided functions over the different paralogs^7,10^. One paralog (Aurora B in humans) is the catalytic centre of the chromosomal passenger complex (CPC), a key regulator of mitotic events at the cell equator, which initially hitchhikes on the inner centromere and finally remains at the central spindle to orchestrate cytokinesis. In addition to the single Aurora kinase, the CPC consists of inner centromere protein (INCENP), Survivin and Borealin^11,12^ that guide its localisation to kinetochores, spindle and the central spindle. It is also critical for mitotic histone phosphorylation, fixing errors in kinetochore-spindle attachment, and correct cytokinesis (Fig. 1b).

In *Plasmodium*, there are also three ARKs, which have divergent sequence and mostly lack canonical scaffolds^10,13,14^. ARK1, 2 and 3 are refractory to gene disruption and indispensable during asexual blood stage schizogony in both *P. berghei* and *P. falciparum*^13–15^. ARK1 associates with the spindle pole body during schizogony^16,17^, but its function is not understood during this process and its location and function in sexual stages remains unknown. Our recent studies on ARK2 revealed its association with spindle microtubules (MTs) forming a divergent protein complex with EB1, myosin K and MISFIT^10^, but little is known of ARK1 and 3 functions during male gametogony or whether they are essential for parasite transmission. ARK1 function has been studied in another apicomplexan, *Toxoplasma gondii*, where it interacts with INCENP1–2 and is important for asexual replication, but its function during male gametogony is difficult to study because the sexual cycle of these parasites only takes place in the gut of cats (Fig. 1b)^18^.

Here, we have investigated *Plasmodium* ARK1, integrating studies of location, function and molecular interactions across two life-cycle stages using *P. falciparum* (Pf, for asexual blood-stage schizogony) and *P. berghei* (Pb, for male gametogony). High-resolution live-cell imaging and expansion microscopy revealed ARK1 associated with the mitotic spindle, kinetochores, and the inner MTOC. Conditional gene knockout using rapamycin-DiCre in *P. falciparum*^19^ and gene knockdown using a promoter-trap approach in *P. berghei*^20^ revealed that ARK1 regulates spindle biogenesis, karyokinesis and cytokinesis during both schizogony and male gametogony, suggesting that ARK1 is a critical regulator of *Plasmodium* mitosis. Interactome analysis also identified *Plasmodium* ARK1 as a component of a non-canonical Chromosomal Passenger Complex (CPC) comprising the two divergent INCENP1 and INCENP2, but without the canonical subunits Survivin and Borealin. These data underscore the evolutionary divergence of the *Plasmodium* mitotic machinery from that of its host, and therefore its potential as a target for therapeutic intervention at multiple stages of the lifecycle.

## Results

### ARK1 is present in asexual stage parasites undergoing mitotic division

**Figure 2:**
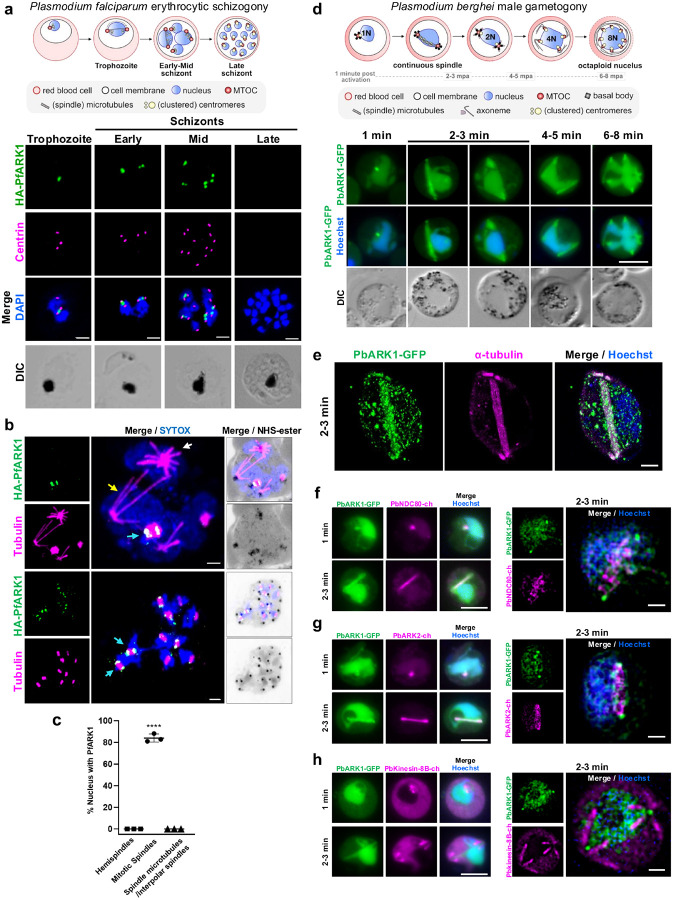
Location of ARK1 during *P. falciparum* asexual division and *P. berghei* sexual male gametogony. **a**. Schematic of asexual division during *P. falciparum* blood stage schizogony. HA-PfARK1-loxP parasites were synchronized and IFA was performed using anti-HA (green) and anti-centrin (magenta) antibody on trophozoites and schizonts. DNA was stained with DAPI. PfARK1 was observed transiently as a punctum only at nuclei with duplicated centrin foci. Scale bar = 2 mm. **b**. Ultrastructure expansion microscopy (U-ExM) was performed on HA-PfARK1-loxP schizonts using anti-HA and anti-tubulin antibodies. Hemispindles (white arrow), mitotic spindles (cyan arrow) and interpolar spindles (yellow arrow) that correspond to different stages of nuclear division were observed. PfARK1-positive puncta were mainly associated with mitotic spindles with the puncta flanking the spindle. Scale bar = 2 mm. **c**. Developing merozoites exhibiting PfARK1 foci (panel b) in association with hemispindles, mitotic spindles or interpolar spindles were quantified across three independent biological replicates (SEM ± SE, one way ANOVA, ****P < 0.0001, n = 3, number of parasites = 40). **d**.Schematic of sexual male gametogony. Live cell images show the location of PbARK1-GFP (green) at different time points (1–8 min) during male gametogony. DNA (blue) was stained with Hoechst. DIC, differential interference contrast. Representative images of more than 50 cells with more than three biological replicates. Scale bar: 5mm. **e**. U-ExM images showing location of PbARK1-GFP (green) and α- tubulin (magenta) detected with anti-GFP and anti-tubulin antibodies, respectively. Hoechst was used to stain DNA (blue). Scale bar = 5 mm. Representative images of more than 10 cells from two biological replicates. **f**. Live cell imaging showing the location of PbARK1-GFP (green) in relation to kinetochore marker (NDC80-ch [mCherry; magenta]) at different time points during gametogony. DNA is stained with Hoechst dye (blue). Merge: green, magenta, and blue images merged. Representative images of more than 20 cells with three biological replicates. Scale bar= 5 mm (left panel). 3D-SIM images of fixed gametocytes at 1.5 min post activation (at the first spindle stage) showing the location of PbARK1-GFP and NDC80-mCherry. Representative image of more than 10 cells from more than two biological replicates. Scale bar = 1 mm (right panel). **g**. Left panels: live cell imaging showing the dynamics of PbARK1-GFP (green) in relation to spindle protein kinase ARK2-mCherry (magenta) at different time points during gametogony. DNA is stained with Hoechst dye (blue). Merge: green, magenta, and blue images merged. Representative images of more than 20 cells with three biological replicates. Scale bar = 5 mm (left panel). Right panels: 3D-SIM images of fixed gametocytes at 1.5 min post activation (at the first spindle stage) showing the location of PbARK1-GFP and ARK2-mCherry. Representative image of more than 10 cells from more than two biological replicates. Scale bar = 1 mm. **h**. Left panels: live cell imaging showing the dynamics of PbARK1-GFP (green) in relation to basal body/axoneme marker kinesin-8B-mCherry (magenta) at two different time points during gametogony. DNA is stained with Hoechst dye (blue). Merge: green, magenta, and blue images merged. Representative images of more than 20 cells with three biological replicates. Scale bar = 5 mm Right panels: 3D-SIM images of fixed gametocytes at 1.5 min post activation (at the first spindle stage) showing the location of PbARK1-GFP and kinesin-8B-mCherry. Representative image of more than 10 cells from more than two biological replicates. Scale bar = 1 mm.

We studied the location of PfARK1 in asexual blood stages, using a rapamycin-inducible dimerizable Cre recombinase (DiCre)-based strategy to knockout the ARK1 gene in the 1G5DC parasite line (Extended Data Fig. 1)^19^. As a first step in this strategy, we generated a HA-PfARK1-loxP parasite line, and these parasites were used to locate ARK1 during parasite division using an antibody to the HA-tag. Indirect immunofluorescence (IFA) indicated that ARK1 is expressed in trophozoite and schizont stages, but not detectable in late schizonts and all developing merozoites (Fig. 2a). ARK1 was present as a punctum proximal to the MTOC also known as a centriolar plaque, as identified by staining with an anti-centrin antibody, present only in cells with two or more associated centrin puncta, indicative of ongoing mitosis (Fig. 2a). To gain a deeper insight into ARK1 dynamics, Ultrastructure Expansion Microscopy (U-ExM) was performed using an anti-tubulin antibody to track spindle biogenesis. As reported previously^21^, parasites possessed hemispindles prior to the onset of division, but PfARK1 was not expressed in these parasites or in parasites containing interpolar spindles (Fig. 2b, c). Strikingly, PfARK1 was specifically found associated with mitotic spindles and PfARK1-positive double puncta seemed to be present at the polar ends of the mitotic spindles (Fig. 2b, c). These data strongly indicate that ARK1 is expressed transiently, and only when parasites undergo mitotic division.

These observations were also validated using the rodent malaria parasite, *P. berghei*, a well-established model for studying gametogony and parasite development within the mosquito host. The subcellular location of PbARK1 in asexual stages was examined by generating an endogenous, C-terminal GFP-tagged PbARK1 (PbARK1-GFP) transgenic parasite line (Extended Data Fig. 2a-c and Supplementary Notes). Live cell imaging of PbARK1-GFP in asexual blood stages revealed a diffuse location in early ring stages. During trophozoite and early schizont stages, PbARK1-GFP re-localised to discrete focal points in the nucleus (Extended Data Fig. 2d). As schizogony progressed, the focal points of fluorescence divided into two closely associated puncta, which followed subsequent asynchronous nuclear divisions and eventually became diffuse during the later stages (Extended Data Fig. 2d, e). These observations are consistent with the ARK1 location in *P. falciparum* as described above (Fig. 2a).

#### PbARK1 is located in the nucleoplasm, at the spindle poles and at microtubules during male gametogony

In male gametocytes, PbARK1-GFP had a diffuse distribution predominantly within the nucleoplasm. Within one-min post-activation this was concentrated to a single discrete focal point adjacent to the DNA (Fig. 2d), and then the distribution rapidly extended to form an arc-like structure, which within three-min post-activation divided into two distinct puncta positioned at opposite sides of the nucleus (Fig. 2d and Supplementary Video 1). The two PbARK1-GFP foci elongated again, forming two arc-like structures along the periphery of the nucleus (Fig. 2d and Supplementary Video 2). By 6- to 8-min post-activation, a subsequent round of extension and division had produced eight discrete PbARK1-GFP foci, coincident with the completion of three successive rounds of mitosis (Fig. 2d and Supplementary Video 3).

The arc-like pattern of PbARK1 observed in live cell imaging coincided with the pattern of anti-tubulin staining in IFA and U-ExM, suggesting an association with the mitotic spindle and spindle poles (Fig. 2e and Extended Data Fig. 2f). To further investigate ARK1 location, dynamics, and association with mitotic spindles and kinetochore segregation, we compared the location of PbARK1 with that of the kinetochore marker NDC80, spindle marker ARK2 and cytoplasmic axonemal protein kinesin-8B. We generated dual fluorescent parasite lines expressing PbARK1-GFP (green) and either PbNDC80-mCherry (magenta), PbARK2-mCherry (magenta) or Pbkinesin-8B-mCherry (magenta) by genetic cross as described previously^4^. Live-cell imaging showed that both PbARK1-GFP and PbNDC80-mCherry were located next to the Hoechst-stained DNA, partially overlapping at the spindle but not at the spindle poles (Fig. 2f left panel, Extended Data Fig. 2g, and Supplementary Video 4). 3D-structured illumination microscopy (SIM) at one-min post-activation showed a clear association of PbNDC80-mCherry with PbARK1-GFP at the spindle but not at the spindle poles (Fig. 2f right panel). In parasite lines expressing PbARK1-GFP and PbARK2-mCherry, these proteins were co-located at the spindle (Fig. 2g left panel, Extended Data Fig. 2h, and Supplementary Video 5). Further 3D-SIM at one-min post-activation showed an association of PbARK1-GFP with PbARK2-mCherry at the spindle, with PbARK1-GFP more outside at the spindle poles (Fig. 2g right panel). However, in a parasite line expressing PbARK1-GFP and Pbkinesin-8B-mCherry (a cytoplasmic marker) there was no overlap between these two proteins (Fig. 2h left panel and Extended Data Fig. 2i), an observation which was further substantiated by 3D-SIM imaging (Fig. 2h right panel).

During meiotic stages of development post-fertilisation, PbARK1-GFP was diffuse in the cytoplasm, with transient nuclear foci forming in zygotes and disappearing in mature ookinetes (Extended data Fig. 3a and Supplementary Notes). A diffuse distribution was also observed throughout oocyst and sporozoite stages, including those from salivary glands (Extended Data Fig. 3b and Supplementary Notes). In liver stages (in HepG2-infected cells), the PbARK1-GFP signal remained diffuse even at 48 hours post infection (h.p.i), with slight perinuclear enrichment at 64 h.p.i (Extended Data Fig. 3c and Supplementary Notes).

### PfARK1 regulates spindle formation and karyokinesis

**Figure 3:**
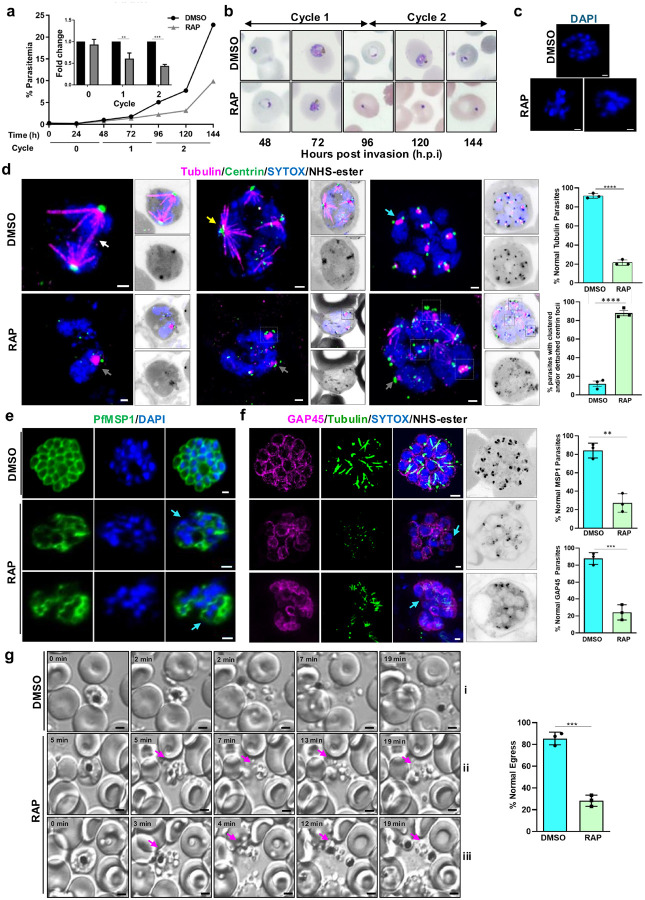
PfARK1 controls karyokinesis, spindle formation and cytokinesis during asexual division. **a**.Growth rate assays were established in the presence of DMSO or RAP with synchronized HA-PfARK1-loxP ring stage parasites. After 24h, DMSO or RAP was washed away, and parasite growth was assessed after each cycle by flow cytometry. *Inset:* fold change in parasitaemia of RAP-treated parasites relative to control (DMSO) (SEM ± SE, Two-way ANOVA, n = 3, **P < 0.01, ***P < 0.001). **b**.HA-PfARK1-loxP parasites treated with either DMSO or RAP as described in panel **a**were smeared, Giemsa-stained, and examined by microscopy at the indicated time point. In the DMSO-treated parasites, the intraerythrocytic developmental cycle progressed as expected, with ring, trophozoite, and subsequent ring stages typical of the 48-hour parasite cycle. However, in RAP-treated, PfARK1-depleted parasites, a significant proportion of parasites had pyknotic morphology. **c**. To assess the impact of RAP on schizont development, HA-PfARK1-loxP parasites treated with DMSO or RAP as described above were treated with E64 to block merozoite egress. Thin blood smears were made at ~40–44 h.p.i in cycle 1 and stained with DAPI. RAP treated parasites contained an unsegmented nuclear mass. Scale bar = 2 mm. **d**. HA-PfARK1-loxP parasites were treated with DMSO or RAP. U-ExM was performed on schizonts (~40–44 h.p.i in cycle 1) during division using anti-tubulin and anti-centrin antibodies, SYTOX and NHS-ester, visualised with magenta, green, blue and inverted greyscale, respectively. In DMSO-treated control parasites, hemispindles (yellow arrow), mitotic spindles (cyan arrow) and interpolar spindles (white arrow) were observed in association with single or double centrin foci. In RAP treated parasites, either speckles of tubulin (grey arrows) or almost complete loss of tubulin staining were observed. In addition, unsegregated nuclei were observed and clusters of centrin were found associated with each nuclear mass (enclosed in squares) Scale bar = 2 mm. U-ExM images are displayed as maximum intensity projection of multiple z slices. Upper Right Panel: parasites with normal tubulin staining reflected by the presence of spindles were counted from U-ExM images (SEM ± SE, unpaired t test, **** P <0.0001, n = 3, number of parasites = 55). Lower Right Panel: % parasites with clustered and/or detached centrin foci (SEM ± SE, unpaired t test, **** P <0.0001, n = 3, number of parasites = 55). **e and f**. HA-PfARK1-loxP parasites were treated with DMSO or RAP as above and cycle 1 schizonts were treated then with E64 for 2 h to block merozoite egress and allow complete maturation and segmentation of the schizonts. IFA (e) or U-ExM (f) was performed on unexpanded (e) or expanded (f) parasites using anti-MSP1 (e) or anti-GAP45 and anti-tubulin (f), respectively. In DMSO treated parasites, clear segmentation was observed whereas RAP-treated parasites either lacked MSP1 and GAP45 staining or contained unsegregated nuclei which were circumscribed by a large MSP1 contour (cyan arrow). U-ExM images are displayed as maximum intensity projection of multiple z slices. Right Panel: Quantification of parasites with normal GAP45 or MSP1 staining was performed (SEM ± SE, unpaired t test, ** P <0.01 *** P <0.001, n=3, number of parasites = 75 for MSP1 and 40 for GAP45).Scale bar = 2 mm. **g**. Time lapse microscopy of HA-PfARK1-loxP parasites cultured in the presence or absence of DMSO or RAP as above and cycle 1 schizonts were anlysed. Selected still images from videos (Supplementary Videos 6–7) are used to illustrate the egress and dispersal of merozoites. DMSO treated parasites egressed from the erythrocytes and dispersed efficiently. RAP treated parasites egressed from erythrocytes but continued to clump-together even after prolonged duration (magenta arrow). Scale bar = 2 mm. Right Panel: parasites undergoing normal egress as defined above were counted (SEM ± SE, unpaired t test, **** P <0.0001, n=3, number of parasites= 110).

The functional role of PfARK1 in asexual blood stage development was assessed by examining the effect of deleting the gene. Rapamycin (RAP) treatment of HA-PfARK1-loxP parasites efficiently excised the floxed sequence (Extended Data Fig. 1c) and caused depletion of PfARK1 protein (Extended Data Fig. 1d). Synchronized ring stage cultures of 1G5DC-SkipFlox (control) (Extended Data Fig. 4a) or HA-PfARK1-loxP parasite lines were treated with RAP or DMSO (control) and parasite growth was monitored every 24 h. In cycle 0 (RAP-treatment) there was little effect on the parasitaemia of HA-PfARK1-loxP parasites. However, a significant growth defect was observed at the beginning of the next cycle (cycle 1) as indicated by a marked reduction in parasitaemia (Fig. 3a and Extended Data Fig. 4b), whilst there was almost no effect of RAP treatment on 1G5DC-SkipFlox (Extended Data Fig. 4a). This growth defect was further accentuated in subsequent cycles. Microscopic examination of Giemsa-stained blood smears suggested normal ring to trophozoite development in cycle 1 DMSO-treated parasites (Extended Data Fig. 4c). However, following RAP treatment and PfARK1 depletion, a significant number of parasites were pyknotic (at ~ 96 hpi) and fewer rings were found in cycle 2 (Fig. 3b). Schizont development following treatment with E64 to block late schizont maturation and merozoite egress was analysed: a significant number PfARK1-depleted cycle 1 schizonts had abnormal morphology, with unsegregated nuclei (Fig. 3c and Extended Data Fig. 4d), which is consistent with the role of PfARK1 in nuclear division. This suggestion, that the decrease in parasitaemia resulting from PfARK1 depletion was due to its role in nuclear division and/or schizont maturation, was investigated further.

To begin to understand how PfARK1 regulates asexual cell division, MTOC duplication and spindle formation were examined. U-ExM revealed the formation of various forms of spindles as described earlier with one or two centrin-labelled foci on the cytoplasmic side of the nuclear envelope (Fig. 3d) and associated with NHS-ester labelled MTOCs or associated with mitotic spindles^21,22^. In PfARK1-depleted parasites there were fewer spindles indicated by either absent or speckled tubulin staining (Fig. 3d and Extended Fig. 4e, grey arrow). As mentioned above, the nuclei appeared unsegregated with associated multiple centrin foci, and centrin aggregates were seen away from the nuclei and disorganized (Fig. 3d and Extended Fig. 4e, square). Collectively, these data suggested that while the MTOC duplication was unaltered, it was disorganized and spindle formation and karyokinesis were impaired in PfARK1-depleted parasites.

### PfARK1 depletion impairs segmentation of daughter merozoites

At the end of schizogony, segmentation of the schizont by invagination of the parasite plasma membrane leads to formation of merozoites prior to their egress. IFAs were performed using antibody to MSP1, a parasite surface marker, to assess segmentation following ARK1 depletion. MSP1 fluorescence with a bunch of grape-like contours, characterized the membrane of control parasites, but in contrast, PfARK1-depleted parasites either lacked MSP1 staining, or contained large aggregations that encompassed multiple merozoites or unsegregated nuclei (Fig. 3e, cyan arrow).

U-ExM of mature (segmented) schizonts with anti-GAP45 antibody-labelling revealed inner membrane complex (IMC) and tubulin labelled subpellicular MT, and rhoptries labeled with NHS-ester (Fig. 3f). However, for the PfARK1-depleted parasites, several unsegregated nuclei were present and these cells either lacked GAP45 staining, or the parasite pellicle (the plasma membrane/IMC) was disorganized (Fig. 3f, cyan arrow). NHS-ester labelled rhoptries were found with some merozoites, typically the ones with GAP45 staining. In some parasites rhoptries were either not formed properly or were absent, which may be due to arrested division of these parasites prior to rhoptry formation. In addition, subpellicular MT were either not observed in the cell or were mislocalised. These observations suggest impaired cytokinesis of daughter merozoites upon PfARK1 depletion.

To study the effect of PfARK1 depletion on merozoite release and egress, time-lapse microscopy was performed^23^. For this purpose, parasites were treated with protein kinase G (PKG)-inhibitor ML-10 to allow schizont maturation but prevent egress, then its removal allowed egress, which was captured by time-lapse microscopy^24,25^. Egress and dispersal of merozoites for control parasites was observed (Fig. 3g and Supplementary Video 6), and while egress from the erythrocyte initially appeared almost normal in PfARK1-depleted parasites, the merozoites either remained attached to the residual body or clumped together (Fig. 3g, magenta arrows, and Supplementary Videos 7, 8) even after prolonged duration. These defects likely result from the abnormal nuclear segregation and cytokinesis in PfARK1-depleted parasites.

### PbARK1 knockdown reveals an essential role during male gametogony and parasite transmission

**Figure 4:**
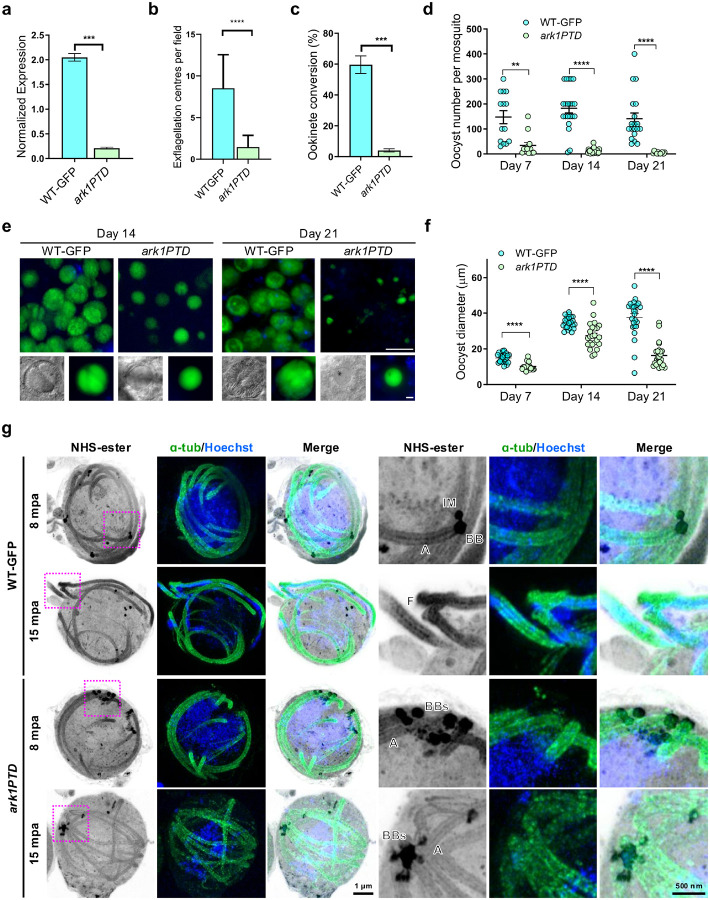
Conditional knockdown of PbARK1 affects male gametogony, disrupting parasite transmission. **a**.qRT-PCR showing normalised expression of PbARK1 transcripts in *ark1PTD* and WT-GFP parasites. Shown is mean ± SEM; n = 3 independent experiments. Student t test was used to examine significant difference. ***p<0.01. **b**. Exflagellation centres per field at 15 min post-activation. n = 3 independent experiments (>10 fields per experiment). Error bar ± SEM. Student t test was used to assess differences between control and experimental groups. Statistical significance is indicated as ***p<0.01. **c**. Percentage ookinete conversion from zygote. n = 3 independent experiments (>100 cells). Error bar ± SEM. Student t test was used to assess differences between control and experimental groups. Statistical significance is indicated as ***p<0.01. **d**. Total number of GFP-positive oocysts per infected mosquito in *ark1PTD* compared to WT-GFP parasites at 7-, 14-, and 21 d post-infection. Mean ± SEM. n = 3 independent experiments. Two-way ANOVA test was employed to assess differences between control and experimental groups. Statistical significance is indicated as **P < 0.01, ***P < 0.001. **e**. Mid guts at 10 × and 63 × magnification showing oocysts of *ark1PTD* and WT-GFP lines at 14-, and 21 days post-infection. Scale bar: 50 mm in 10 × and 20 mm in 63 ×. **f**. The diameter of GFP-positive oocysts in *ark1PTD* compared to WT-GFP parasites at 7-, 14-, and 21 d post-infection. Mean ± SEM. n=3 independent experiments. Two-way ANOVA test was employed to assess differences between control and experimental groups. Statistical significance is indicated as **P < 0.01, ***P < 0.001. **g**. Expansion microscopy images of wild-type-GFP (WT-GFP) and *ark1* gene-knockdown (*ark1PTD*) male gametocytes at 8 min or 15 min post-activation (mpa). The three images on the left show maximum intensity projections of whole-cell z-stack images labelled with NHS-ester (grey), a-tubulin (green) and Hoechst (blue). The three images on the right show magnified views of the areas enclosed by the magenta squares. BB: basal body, BBs: basal bodies (clumped), IM: inner MTOC, A: axoneme. The scale bars are scaled by an expansion factor of 4.954, calculated from a comparison of axoneme diameters measured using expansion and transmission electron microscopy images.

Previous analysis by gene disruption had shown that PbARK1 is probably essential for asexual blood-stage development^14^. To be able to manipulate its expression in sexual stages we used a promoter trap strategy, replacing the *ark1* promoter with the promoter from *ama1*, resulting in *P*_*ama1*_*ark1* hereafter referred as (*ark1PTD*), a gene which is not transcribed in gametocytes but is highly expressed in asexual blood stages (Extended Data Fig. 5a)^26^. Correct genetic integration in the transgenic parasite line was confirmed by PCR (Extended Data Fig. 5b), and *ark1* transcription was shown to be downregulated in *ark1PTD* gametocytes by qRT-PCR (Fig. 4a). A phenotypic analysis of these *ark1PTD* parasites was then performed at different stages of parasite development within the mosquito.

Phenotypic analysis revealed that male gametogony (as scored by exflagellation centres) was severely affected in the *ark1PTD* line compared to GFP control (wild-type-GFP; WT-GFP) parasite (Fig. 4b)^27^, and therefore significantly fewer ookinetes (differentiated zygotes after fertilisation) were formed (Fig. 4c). In mosquitoes fed with *ark1PTD* parasites, we observed significantly fewer oocysts in the midgut at day 7 post infection, in contrast to the hundreds of oocysts in mosquitoes fed WT-GFP parasites (Fig. 4d). The number of *ark1PTD* oocysts was further reduced by day 14 and day 21 post infection compared to WT-GFP oocysts (Fig. 4d). The *ark1PTD* oocysts were also significantly smaller than the WT-GFP oocysts 7 days post infection and diminished further by day 21 post infection (Fig. 4e, f). The number of salivary glands sporozoites was significantly reduced in *ark1PTD* parasites on day 21 (Extended Data Fig. 5c). To further confirm the absence of sporogony, mosquitoes that had been fed with *ark1PTD* or WT-GFP parasites were allowed to feed on susceptible naïve mice. Mosquitoes infected with WT-GFP parasites successfully transmitted the parasite, with blood-stage infection detected in naïve mice after 4 days. In contrast, *ark1PTD* parasites failed to transmit in most experiments (five out of eight) (Extended Data Fig. 5d). However, on three occasions, they were able to establish infection in bite-back experiments, although the prepatent period was markedly prolonged, with blood-stage parasites first detected 14 days post-infection (Extended Data Fig. 5d).

To investigate the effect of *Pbark1* downregulation at the genome wide level, we performed RNA-seq analysis in triplicate, starting with total RNA extracted from purified *ark1PTD* and WT-GFP gametocytes at two time points: before activation (non-activated, 0 min) and at 30 min post-activation (mpa). Differences in gene expression between the correspondent samples were detected. In non-activated gametocytes, only a small number of differentially expressed genes were identified in the *ark1PTD* sample, with 10 genes up- and 8 genes downregulated, respectively (Extended Data Fig. 5e and Supplementary Table 1). Those genes included several fam a and b proteins known to be highly variable during life cycle progression due to their involvement in host immune response modulation^28^. They were therefore not considered as significant. At 30 mpa, 23 genes were upregulated including several conserved *Plasmodium* proteins with unknown function as well as PBANKA_1206900, a tubulin beta chain and putatively critical during gametocyte formation^29^. A significantly higher number of genes (229) were downregulated (Extended Data Fig. 5f and Supplementary Table 1). These genes included several protein kinases, e.g. PBANKA_1228800 and calcium-dependent protein kinase 5 (CDPK5 – PBANKA_1351500), as well as genes involved in lipid biosynthesis including PBANKA_0201300, a putative lysophospholipase, PBANKA_1127000, and a choline/ethanolaminephosphotransferase. Also, down-regulated were myosin essential light chain (ELC) and skeleton-binding protein 1 (PBANKA_1101300), as well as several enzymes known to be critical for sexual differentiation. Gene ontology (GO) analysis also revealed that several genes detected as downregulated were associated with ribosome biogenesis (Extended Data Fig. 5g). These data indicate not only a potential cell cycle arrest but also most likely stress sensing and translational inhibition as a result of PbARK1 depletion.

#### ark1PTD parasites have defective spindle formation, MTOC separation, and axoneme formation during male gametogony

To further investigate the function of PbARK1 during male gametogony, WT-GFP and *ark1PTD* male gametocytes were observed during gametogony using U-ExM. In WT-GFP male gametocytes at 8 mpa, individual MTOCs were distinct and separated, whereas in *ark1PTD* male gametocytes multiple MTOCs were clumped and aggregated together (Fig. 4g and Extended Data Fig. 6a). At 15 mpa, WT-GFP male gametocytes were observed undergoing exflagellation, whereas in *ark1PTD* male gametocytes the MTOCs were still clumped together and no exflagellation was observed (Fig. 4g and Extended Data Fig. 6a).

**Figure 5:**
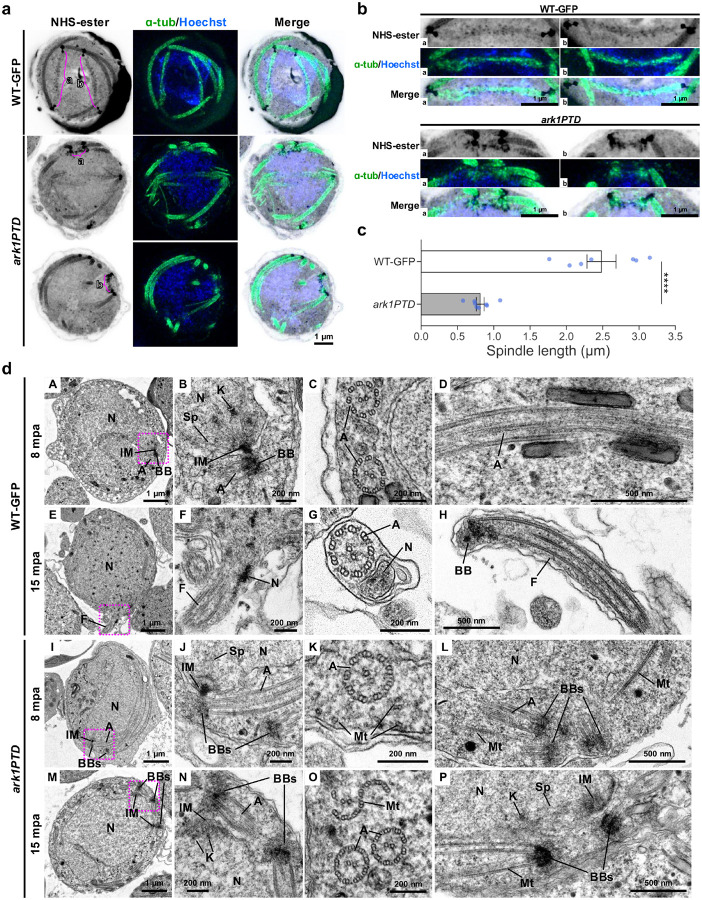
*Pbark1*gene-knockdown disrupts spindle and axoneme formation. **a**.Expansion microscopy images of *P. berghei* wild-type-GFP (WT-GFP) and *ark1*gene-knockdown (*ark1PTD*) male gametocytes at 8 min post-activation (mpa). The maximum intensity projections of entire cells from a z-stack, containing spindles. Cells were labelled with NHS-ester (grey), a-tubulin (green) and Hoechst (blue). Spindles are indicated by magenta lines with a and b. **b**. Magnified views of each spindle of WT-GFP and *ark1PTD*highlighted with magenta lines with a and b in Fig. 5a. **c**. Comparison of spindle lengths measured from expansion microscopy data of WT-GFP and *ark1PTD*. Data represent mean ±SEM. (n = 7 spindles for WT-GFP and 8 spindles for *ark1PTD*, respectively across 3 different cells). Student t test was used to examine significant difference. ****p<0.0001. The scale bars and spindle lengths are scaled by an expansion factor of 4.954, calculated from a comparison of axoneme diameters measured using expansion and transmission electron microscopy images. **d**.Transmission electron microscopy images of WT-GFP and *ark1*gene-knockdown male gametocytes at 8 min or 15 min post-activation. b, f, j and n are magnified views of the areas enclosed by the magenta squares in a, e, i and m, respectively. N: nucleus, IM: inner spindle MTOC, BB: basal body, A: axoneme, K: kinetochore, Sp: spindle microtubule, F: flagellum, BBs: basal bodies (clumped), Mt: microtubule.

We examined whether the lack of MTOC separation in the *ark1PTD* line led us to examine whether this was caused by a defect in spindle formation. At 8 mpa, long spindles with clearly separated MTOCs were present in WT-GFP parasites, but in *ark1PTD* lines, short spindles, with the two MTOCs remaining close together (Fig. 5a, b) were present. Measurement showed that *ark1PTD* spindles were significantly shorter (~ 0.8 μm) than WT-GFP spindles (~ 2.5 μm; Fig. 5c). These findings corroborated studies on PfARK1 during schizogony as depletion of the kinase resulted in abnormal spindle formation and accumulation of MTOCs around unsegregated nuclei (Fig. 3d).

TEM observations further revealed structural defects in *ark1PTD* male gametocytes. At 8 mpa, in the *ark1PTD* line, MTOCs formed a normal bipartite structure consisting of an inner spindle MTOC and an outer basal body MTOC, similar to those in WT-GFP male gametocytes, and normal axoneme formation was observed (Fig. 5dA–D, I–L and Extended Data Fig. 6bA-D), However, we also observed MTOCs that appeared detached from the nucleus in the *ark1 PTD* line (Fig. 5dI, J and Extended Data Fig. 6bA, B, D). As revealed by expansion microscopy, multiple basal body MTOCs were often aggregated and clumped together (Fig. 5dL). Within the cytoplasm, we observed singlet or doublet MT that may have been axoneme precursors and likely represent failures in axoneme formation (Fig. 5dK, L).

At 15 mpa, WT-GFP gametocytes had started to form gametes with flagellum and nucleus (Fig. 5dE, F, G, H), while in the *ark1PTD* line there was incomplete axoneme formation and exflagellation rarely occurred (Fig. 5dM–P and Extended Data Fig. 6bE-H). In addition, two basal body MTOCs remained close together without proper separation (Fig. 5dM, N, P), with short spindle MT visible between them (Fig. 5dP and Extended Data Fig. 6c).

### PbARK1 is part of a divergent chromosome passenger complex (CPC) with two INCENP proteins

**Figure 6:**
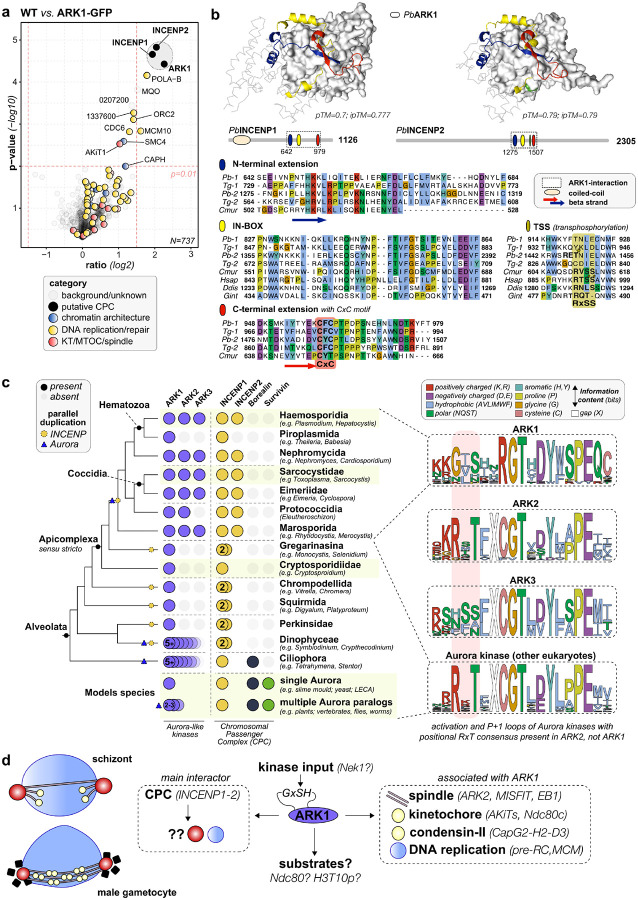
PbARK1 is the divergent Aurora kinase of the alternative chromosomal passenger complex (CPC) in *Plasmodium* spp. **a**. Volcano plot of GFP-trap pulldown followed by LC-MS/MS analysis for ARK1-GFP from both 8–9 hr schizont and 1.5-min male gametocyte lysates. **b**. Zoom-in on the domain/motif topology of INCENP1/2 in apicomplexans (Pb = *P. berghei*; Tg = *T. gondii*; Cmur = C. *muris*) and model organisms (Hsap = *Homo sapiens*; Ddis = *Dictyostelium discoideum*; Gint = *Giardia intestinalis*), focused on the extended IN-box region (blue-yellow-red) that interacts with the kinase domain of ARK1. Arrows indicate an antiparallel beta sheet formed by the N- and C-terminal extensions of the IN-box. Green: TSS transphosphorylation site (asterisks indicate (putative) phosphorylated sites) in eukaryotic INCENP homologs, but not in INCENP1/2 in *P. berghei* (Pb) and *T. gondii* (Tg). RET is a candidate transphosphorylation site in PbINCENP2 (Pb-2). Protein structures are predicted using AlphaFold3, for aligned error plot, see Extended Data Fig. 8. **c**.Presence/absence of ARK1/2/3, and components of the chromosomal passenger complex amongst the model eukaryotes, the SAR supergroup with a specific focus on Apicomplexa. Right: consensus sequence logos of the activation and P+1 loop of Aurora kinases in Clustal color scheme: divergent phosphorylation consensus in ARK1 (GxSH instead of RxT). **d**. Summary of findings and discussion points from this study.

To explain the ARK1 phenotypes and confirm the logic of the observed subcellular localisations, we wanted to understand what protein scaffolds or modulator proteins might direct ARK1 activity and localisation during both asexual and sexual stages of the parasite. We therefore prepared lysates of *P. berghei* schizonts and male gametocytes from PbARK1-GFP and WT-GFP control parasites, immunoprecipated the protein with GFP-Trap and then identified bound proteins using mass spectrometry. At both stages we identified two divergent inner centromere proteins (INCENP1/2) as major ARK1 interactors (Fig. 6a and Extended Data Fig. 7). Although the differences were not significant (n = 2 for each sample), in the male gametocyte sample we observed co-variation of various spindle and kinetochore proteins amongst the pulldowns (Supplementary Table 2): for example, the putative ARK2-based spindle complex including MISFIT-MyoK-EB1, several kinetochore subunits e.g. Apicomplexan Kinetochore proteins (AKiTs) 1, 3, 5 and 7 and the condensin-II-specific subunits CapH2-G2-D3. Strikingly, we saw no survivin/borealin-like proteins in the interactome, confirming earlier predictions that *Plasmodium* spp., and more generally apicomplexans, lack other conserved components of the CPC (Fig. 6c). Since the spatiotemporal dynamics of ARK1 suggest that it may not act as the classic passenger complex on chromosomes, it is possible that the chromatin-directing function of Survivin and Borealin has become obsolete for apicomplexans and that the structural motifs for localisation and activation of ARK1 by INCENP1 and 2 might be revealed upon detailed comparative sequence analyses.

*T. gondii*, another apicomplexan, sister to *Plasmodium* spp. also has two INCENP paralogs that interact with TgARK1, so we sought to reconstruct the evolutionary history of the INCENP proteins with a focus on alveolates and apicomplexans (Fig. 6b, c). The duplication that gave rise to INCENP1 and INCENP2 can be traced back to the common ancestor of all Apicomplexa (sensu stricto). We found parallel but separate duplications in other different alveolate lineages such as apicomplexpan-like Squirmida, Chrompodellida and dinoflagellate-related lineages concomitant with the loss of borealin/survivin (Fig. 6c), suggesting INCENP duplications might compensate for loss of these chromatin-directing subunits. Interestingly, INCENP2 is absent from Piroplasmida, lineages which have also lost ARK2 and ARK3, suggesting that INCENP2 might also play a role in ARK2/3-based regulatory pathways. A co-fold model of *P. berghei* INCENP1/2 and ARK1 using AlphaFold3, suggests that the conserved IN-box region involved in kinase interaction is flanked in all apicomplexan proteins by N- and C-terminal extensions that form a beta-sheet – folding INCENP1/2 around ARK1 (Fig. 6b and Extended Data Fig. 8). In addition, the C-terminal extension has a conserved CxC motif, possibly involved in posttranslational modification (Fig. 6b). We could only predict a coiled-coil in the N-terminus of INCENP1, and no further structural features could be discerned for INCENP2 using AlphaFold3. Such absence of any feature makes it hard to predict the binding mode of INCENP2 either as a single ARK1-INCENP2 interaction or forming an INCENP2-ARK1-INCENP1 trimeric complex.

Finally, we sought whether any non-canonical ARK1/INCENP features might indicate alternative CPC functions in *Plasmodium* spp., such as the highly conserved autophosphorylation sites in both Aurora B and INCENP, which are crucial for the kinase activity of the CPC in vertebrates^30^. We found that both the broadly conserved autophosphorylation site in the activation loop of Aurora kinases (RxTxCGT) and the activating phosphosite on INCENP (RTSS in human) are markedly different (GxSH in ARK1) and (RTSS absent from INCENP1/2). However, some Aurora consensus sites can be found in the vicinity among INCENP1/2 paralogs (Fig. 6b – green boxes). ARK2 paralogs have a conserved Aurora activation loop, while ARK3 has a serine-rich activation loop with multiple candidate activating phosphorylation sites (Fig. 6c). The absence of clear conserved auto/trans-phosphorylation sites between ARK1 and INCENP1/2 in *Plasmodium* spp. indicate CPC kinase activity might be regulated in a different fashion, possibly by input of another kinase.

## Discussion

Our cross-stage analysis identified ARK1 as a central organiser of *Plasmodium* mitosis using *P. falciparum* and *P. berghei* model systems. First, ARK1 localises to the spindle and inner MTOC specifically during mitosis, and its depletion in *P. falciparum* reduces spindle formation, prevents proper karyokinesis, disrupts segmentation and impedes egress. Second, during the rapid mitoses of male gametogony, *P. berghei* ARK1 dynamics follow spindle poles; knockdown shortens spindles, block MTOC separation and axoneme biogenesis, and abolishes transmission. These phenotypes position ARK1 upstream of both chromosome segregation and gamete formation.

Our findings show that ARK1 is preferentially expressed and targeted during the mitotic stages marked by spindle formation. Live cell imaging and expansion microscopy revealed that ARK1 locates to the spindle apparatus and the spindle poles, corresponding to the inner core of the bipartite MTOC^4,21^. Expansion microscopy using centrosome (centrin) and microtubule (tubulin) markers confirmed that ARK1 associates with spindle poles within the inner MTOC in both mitotic stages. Some of these features had been observed earlier for *P. falciparum* ARK1 and further it had been shown that its location is limited to mitotically dividing stages^17^. Dual fluorescence microscopy analysis during male gametogony revealed that it colocalised with ARK2, another aurora kinase reported recently, and the kinetochore marker NDC80 at the plus ends of spindle microtubules, suggesting a cooperative role for ARK1 and ARK2 at equatorial spindles. It also has a role in kinetochore dynamics, as observed previously for ARK2^10^. However, live cell imaging in *P. berghei* clearly showed that it is part of the inner MTOC as it does not colocalise with the basal body and axoneme marker, kinesin-8B. This contrasts with *T. gondii* ARK1, which, when tagged at the N-terminus, exhibited a dynamic localisation to both nucleus and cytoplasm during division, but remained predominantly nuclear in non-dividing cells^18,31^. Its colocalisation with kinetochore NDC80 and ARK2 markers further supports a dual role in spindle organization, kinetochore segregation, and spindle pole integrity—reminiscent of Aurora B, which localises to centromeres, the central spindle, and the spindle midzone^5,6,32,33^.

Functional genetic studies in *P. falciparum* and *P. berghei* had indicated that ARK1 is likely essential in asexual blood stages^13,14^. Attempts to generate conditional knockdowns using the DD/Shield system in *P. falciparum* were unsuccessful, as reported recently^17^. Here using the DiCre-Rapamycin system for *P. falciparum*^19^ and promoter trap strategy for *P. berghei*^20^, we demonstrate that ARK1 depletion leads to defects in spindle formation and karyokinesis. In *P. falciparum*, ARK1 is required for proper spindle elongation, parasite growth, cytokinesis and egress. In *Toxoplasma*, overexpression of a kinase dead mutant of TgARK1 disrupts chromosome segregation and spindle pole body maturation, similar to some of the division defects observed here in asexual stages, although the mechanism was unexplored^18,31^.

During male gametogony, ARK1 knockdown led to defects in the spindle apparatus and failure of MTOC separation, suggesting its associated function with the outer basal body^4^. This was accompanied by abnormal axoneme formation and very few flagellated male gametes, indicating a failure in MTOC-basal body segregation. These results suggest that while ARK1 is part of the inner MTOC, its functional association with the outer MTOC is essential for successful mitosis and gamete formation, similar to the egress and spindle elongation defects seen in *P. falciparum*. These features also show some resemblance to those of Aurora B in many model systems^5,6,32,33^.

Our protein interactome analysis reveals that ARK1 is part of a non-canonical chromosomal passenger complex (CPC), comprising two divergent INCENP homologs and lacking the canonical chromatin-targeting components Borealin and Survivin. This is consistent with earlier molecular cell biological studies in *Toxoplasma*^18^. While studies in *Toxoplasma* also reported two INCENPs, they did not detect the presence or absence of other CPC components. The lack of chromatin-targeting components might suggest that INCENP1/2 can facilitate such interactions themselves or simply mean these are lost and replaced by interactions of different proteins. For instance, INCENPs in apicomplexans may form alternative CPC complexes, similar to *Trypanosoma*, where INCENP associates with kinesin-1A/B^34^. A potential candidate kinesin might be kinesin-8X, which shows a similar localisation pattern during different life stages as ARK1^35^. Interestingly, plants and heterokont algae also harbour divergent Survivin-like proteins, with a central conserved but highly divergent helix suggesting a possible broader evolutionary divergence of CPC components across eukaryotes^36^. Future studies will need to include a further scrutiny of INCENP1/2 localisation and interactomes to determine whether any new CPC components might be present in apicomplexans.

Comparative genomics provides an evolutionary scenario for this atypical CPC. INCENP duplication into INCENP1 and INCENP2 likely arose in the apicomplexan ancestor, concomitant with the loss of Survivin and Borealin (Fig. 6). Notably, INCENP2 has been secondarily lost in piroplasmids, which also lack ARK2 and ARK3, hinting at functional coupling between Aurora paralogs and specific INCENP variants. Structural predictions using AlphaFold3 suggest that apicomplexan INCENPs contain extended IN-box regions that fold around the ARK1 kinase domain and feature a conserved CxC motif absent from other eukaryotic homologs. At the same time, ARK1 itself shows a divergent activation loop (GxSH instead of the canonical RxT), and INCENPs lack the canonical transphosphorylation site (TSS) site found in other eukaryotes. Such changes might indicate that other kinases are necessary to activate ARK1. Together, these features indicate that apicomplexans have likely rewired CPC architecture and regulation, replacing canonical chromatin-targeting logic with INCENP-centric scaffolds that localise ARK1 at the inner MTOC, spindle and nucleoplasm.

While the function of the other two ARKs-ARK2 and ARK3- is unclear, at least in asexual stages, our recent studies on PbARK2 have provided insights into its role in sexual transmission stages. While ARK2 regulates spindle dynamics during male gametogony just like ARK1 in the present studies, surprisingly, its deletion did not impact exflagellation, unlike that of the ARK1 mutant (Fig. 4b). Previous studies also revealed that PbARK2 deletion caused an increase in the expression of ARK1 and ARK3^10^. Therefore, in the light of the present work, it is reasonable to suggest that ARK1 and ARK2 may have some overlap of function during male gametogony, particularly in spindle biogenesis. It was also interesting to note that the deletion of PfARK1 resulted in higher PfARK2 levels in schizonts (Extended Data Fig. 1f), although this increase was insufficient to complement the PfARK1 mutant. While there may be some overlap in ARK1 and ARK2 function, each is indispensable for parasite survival.

Overall, this study suggests that *Plasmodium* ARK1 regulates spindle elongation during mitotic division and associates at the inner nuclear pole of the MTOC. However, its association with the outer MTOC is essential for karyokinesis and for flagellated gamete formation. ARK1 appears to represent a unique Aurora kinase paralog that, while divergent in structure, shares functional similarities with Aurora B in humans. This suggests that *Plasmodium* has evolved an atypical mechanism of cell division, where many of the canonical molecules are either missing or highly divergent, reflecting the parasite’s adaptation to complex life cycle transitions.

## Methods

### P. falciparum cultures

*Plasmodium falciparum* strain 1G5DC was obtained from European Malaria Reagent Repository (EMRR) and DSM1 was obtained from *BEI* resources, Malaria Research and Reference Reagents Resource Centre (MR4), American Type Culture Collection (ATCC). Parasites were maintained in O + human erythrocytes (5% hematocrit) in RPMI-1640 supplemented with 0.5% Albumax II and 50 μg/mL hypoxanthine^37^. Cultures were maintained at 37°C under a mixture of 5% CO_2_, 3% O_2_, and 91.8% N_2_ or 5% CO_2_. Fresh erythrocytes and culture medium were used to dilute parasites to maintain 3–5% parasitaemia and 5% haematocrit. Relevant drugs were used during the culture of various transgenic lines, as described below. Parasite synchronization was carried out using 5% sorbitol as described previously^38^.

#### Generation of transgenic parasite lines

All PCR primers used in this study were synthesized by Sigma and are indicated in Supplementary Table 3.

### 1G5DC SkipFlox

A dimerisable Cre recombinase (diCre) expressing parasite line was generated by transfecting pSkipFlox plasmid, a kind gift from Tobias Spielmann (Addgene Plasmid #85797) into the parent line 1G5DC^19^. Parasites were selected in the presence of 2.5 μg/ml of blasticidin.

#### pSLI-HA-ARK1- loxP

To generate a conditional knockout transgenic parasite, a selection-linked integration (SLI) approach was used. To generate the construct, a 592 bp homology region corresponding to the 5’-end of the PfARK1 gene was amplified using primers (P1 and P2) derived from 3D7 genomic DNA. The PCR product was cloned into *NotI* and *PmeI* sites upstream of loxP and a Myc-tag coding sequence in the pSLI-N-sandwich-loxP (K13) vector, which was a gift from Tobias Spielmann (Addgene Plasmid #85793). A recodonised sequence version of full-length PfARK1 with a N-terminal 3xHA tag and followed by a loxP site was custom synthesised as a G-block (Genscript). The G-block was cloned in the above construct downstream of yDHODH using *NheI* and *XhoI* restriction sites^19^.

### P. falciparum transfection

The pSLI-HA-PfARK1-loxP construct was transfected in 1G5DC SkipFlox parasites at the ring stage using ~ 100μg of purified plasmid DNA. Transgenic parasites were selected by using 2nM WR99210 and integrants were then enriched using 1.5μM DSM-1. The parasites that appeared after drug selection were genotyped using PCR primers (Supplementary Table 3 and Extended Data Fig. 1a). The desired integration was confirmed by sequencing of PCR products.

#### Conditional knockout of PfARK1

HA-PfARK1-loxP parasites were treated with 250 nM rapamycin at ring stage for 24 hours. The genomic DNA was isolated from DMSO/RAP-treated parasites and excision of the PfARK1 locus flanked by loxP sites in the transgenic parasite was confirmed by PCR using primers (P7/P8).

### P. falciparum growth rate assays

HA-PfARK1-loxP parasites were cultured in the presence of 10 nM WR99210, 2.5 μg/ml blasticidin and 1.5μM DSM-1. Cultures were maintained at 2% hematocrit and 7–8% parasitaemia. To perform the growth rate assay, 1G5DC-SkipFlox and HA-PfARK1-LoxP parasites were synchronised at ring stage for two cycles using 5% sorbitol for 20 min at 37°C, and the rings were seeded at 0.5% parasitaemia and 2% hematocrit. To deplete ARK1, parasites were treated with either DMSO (vehicle control) or 250 nM rapamycin for 24 h. Thin Giemsa-stained blood smears were examined periodically under a microscope to quantify the parasitaemia at different parasite stages. For assessment of parasitaemia by flow cytometry, cells were fixed by incubation in a solution containing 1% paraformaldehyde and 0.0075% glutaraldehyde at ambient temperature for 15 min on an end-to-end rocker. Post-fixation, samples were either stored at 4°C or stained immediately with Hoechst 33342 dye at 37°C for 10 min. After staining, samples were washed with FACS buffer and then analysed on either BDverse (BD Biosciences) or BD FACSymphony A1 Flow Cytometers, acquiring between 10,000 and 100,000 events per sample. Data analysis was carried out using FlowJo software version 10.10.0.

### Ethics statement (Studies on P. berghei)

All animal procedures were approved following an ethical review process and conducted in accordance with the United Kingdom Animals (Scientific Procedures) Act 1986, under Home Office Project Licenses (PDD2D5182 and PP3589958). Female CD1 outbred mice (6 to 8 weeks old) were obtained from Charles River Laboratories and used for all experiments carried out in the UK.

### Purification of P. berghei schizonts and gametocytes

Blood-stage parasites were obtained from infected mice on day 4 post-infection and cultured at 37°C with gentle rotation (100 rpm) for 11 or 24 hours. Schizonts were purified the following day using a 60% v/v NycoDenz gradient prepared in phosphate-buffered saline (PBS). The NycoDenz stock solution consisted of 27.6% w/v NycoDenz in 5 mM Tris-HCl (pH 7.2), 3 mM KCl, and 0.3 mM EDTA.

Gametocyte purification was performed by first infecting phenylhydrazine-treated mice^39^, followed by sulfadiazine treatment two days post-infection to enrich for gametocytes. Blood was collected on day 4 post-infection, and gametocyte-infected cells were purified using a 48% v/v NycoDenz gradient in PBS, prepared from the same NycoDenz stock solution. Gametocytes were collected from the interface and subsequently activated for downstream assays^40^.

### Generation of P. berghei transgenic parasites

GFP-tagging constructs were generated using the p277 plasmid vector and transfected into parasites as previously described^41^. A schematic representation of the wild-type *ark1* locus, the GFP-tagging constructs, and the recombined *ark1* locus is shown in Extended Data Fig. 2a. Correct integration of the *gfp* sequence at the *ark1* locus was confirmed by diagnostic PCR using primer 1 (intArk1tg) and primer 2 (ol492), as indicated in Extended Data Fig. 2b. The expression of native ARK1-GFP was confirmed by Western blot analysis (Extended Data Fig. 2c).

To investigate the function of ARK1, a promoter trap strategy was employed using double homologous recombination to generate a conditional knockdown line (*ark1PTD*). The knockdown construct was derived from the P*ama1* plasmid (pSS368), in which *ark1* was placed under the control of the *ama1* promoter, as previously described^20^. A schematic representation of the endogenous *ark1* locus, the targeting constructs, and the recombined *ark1* locus is shown in Extended Data Fig. 5a. Diagnostic PCR was performed to confirm successful integration, as outlined in Extended Data Fig. 5a. Integration at the 5′ locus was verified using primers intPTD31_5 (Primer 1) and 5′-intPTD (Primer 2), while integration at the 3′ end was confirmed using primers intPTD31_3 (Primer 3) and 3′-intPTama1 (Primer 4) (Extended Data Fig. 5b). Primer sequences are listed in Supplementary Table 3. Transfections were carried out by electroporation using *P. berghei* ANKA line 2.34 for GFP-tagging experiments and ANKA line 507cl1 expressing GFP for generation of the knockdown line^27^.

### Generation of dual tagged parasite lines (P. berghei)

To generate dual-labelled parasites, ARK1-GFP parasites were mixed in equal proportions with either NDC80-mCherry, ARK2-mCherry, or kinesin-8B-mCherry labelled parasites and co-injected into mice. Four to five days post-infection, when gametocytaemia was high, Anopheles mosquitoes were allowed to feed on the infected mice. Mosquitoes were examined for oocyst development and sporozoite formation at days 14 and 21 post-feeding. Infected mosquitoes were then used to infect naïve mice via mosquito bite-back. Blood-stage infection was assessed 4–5 days later by Giemsa-stained blood smear microscopy. In this way, parasites expressing both ARK1-GFP and either NDC80-mCherry, ARK2-mCherry, or kinesin-8B-mCherry were obtained. Gametocytes from these mixed lines were purified, and fluorescence microscopy was performed as described below to examine protein co-localisation.

### P. berghei phenotype analyses

Infections were initiated by intraperitoneal injection of approximately 50,000 *P. berghei* parasites from WT-GFP or *ark1PTD* lines into mice. Asexual stages and gametocyte production were monitored on Giemsa-stained thin smears. Four to five days post-infection, exflagellation and ookinete conversion were assessed using a Zeiss AxioImager M2 microscope with an AxioCam ICc1 digital camera. For mosquito transmission assays, 30–50 *Anopheles stephensi* (SD 500) mosquitoes were allowed to feed for 20 min on anaesthetised, infected mice with ~ 15% asexual parasitaemia and comparable gametocyte levels (as determined by Giemsa-stained smears). To evaluate midgut infection, ~ 15 mosquito guts were dissected on day 7, 14 and 21 post-feeding, and oocysts were counted using a 63× oil immersion objective on the same microscope setup. Sporozoites in oocysts were counted on day 14 and day 21 post feeding. Salivary glands were isolated on day 21 to count the sporozoites. Bite-back experiments were performed 21 days post-feeding using naïve mice, and blood smears were examined after 3–4 days. All experiments were conducted at least three times to assess the phenotype.

#### Immunofluorescence Assay (IFAs)

*P. falciparum* - Immunofluorescence assays (IFA) were conducted on thin blood smears following a previously described protocol^42^. In brief, air-dried smears were fixed for 2 min using ice cold 1:1 mixture of methanol and acetone. The smears were dried to remove any traces of methanol/acetone followed by blocking with 3% BSA for 45 minutes at room temperature. Samples were incubated with primary antibodies for 12 h at 4°C. After thorough washing with 1X PBS, Alexa Fluor-conjugated secondary antibodies were added and incubated for 2 h at room temperature. Smears were then mounted with VectaShield medium (Vector Laboratories Inc.) containing DAPI for nuclear staining. Microscopy was performed using a LSM980 AR confocal microscope (Carl Zeiss) or Zeiss Axio Observer microscope and image processing was carried out using Zeiss ZEN Blue (versions 3.1 or 3.9). Representative z-stack images were selected for figure preparation unless indicated otherwise. IFA or U-ExM (described below) with MSP1 or GAP45 antibodies was performed to assess segmentation in schizonts that had been treated with 10 μM E64 at ~ 42 h.p.i (cycle 1) for 4–5 hours to prevent egress and allow complete maturation.

*P. berghei* - Purified gametocytes were fixed in 4% paraformaldehyde in microtubule-stabilising buffer on poly-L-lysine–coated slides (Sigma), permeabilised with 0.1% Triton X-100 in PBS, and blocked with 10% goat serum and 3% BSA in PBS. Primary antibodies—rabbit anti-GFP (1:250, Thermo Fisher) and mouse anti–α-tubulin (1:1000, Sigma)—were diluted in 1% BSA and 10% goat serum in PBS and incubated for 1 h. Alexa Fluor 488 anti-rabbit and Alexa Fluor 568 anti-mouse secondary antibodies (1:1000, Invitrogen) were applied for 45 min in the dark. Slides were washed with PBS between steps, mounted with Vectashield containing DAPI (Vector Labs), and sealed. Images were acquired using a Zeiss Axio Imager M2 microscope with a 63× oil immersion objective and AxioCam ICc1 camera. Fluorescence was adjusted using Axiovision (Rel. 4.8) software. Experiments were repeated at least three times, and 20–30 cells per stage were analysed. See figure legends for additional details.

### P. falciparum live-cell imaging

Live imaging was carried out with minor modifications to previously described protocols^25^. HA-PfARK1-loxP parasites were synchronized at the ring stage and treated with either DMSO or 250 nM rapamycin for 24 hours. Schizonts (cycle 1) were subsequently purified and co-cultured with fresh erythrocytes at 2% hematocrit in RPMI1640 complete medium in the presence of 25 nM PKG inhibitor ML10^23,24^. ML10 was removed after 4–6 hours and parasites were plated onto μ-slide, 2-well ibiTreat chambers (ibidi, 80286) pre-coated with 0.5 mg/mL concanavalin A. The slides were maintained at 37°C in a humidified chamber with 5% CO_2_. Imaging was performed using a Zeiss Axio Observer microscope, capturing frames every 3 seconds for a minimum duration of 30 min and the acquired time-lapse videos were processed and analyzed using Axio Vision software (version 4.8.2) or ZEN blue 3.1 or 3.9.

### P. berghei live cell imaging

To examine PbARK1-GFP expression during erythrocytic development, parasites cultured in schizont medium were imaged at various stages, including ring, trophozoite, schizont, and merozoite. Purified gametocytes were assessed for GFP expression and localisation at multiple time points (0 and 1–15 min) following activation in ookinete medium. For nuclear staining, Hoechst dye was used during live-cell imaging. Zygote and ookinete stages were analysed over a 24-hour culture period. Images were acquired using a Zeiss Axio Imager M2 microscope equipped with a 63× oil immersion objective and an AxioCam ICc1 digital camera (Carl Zeiss) using autoexposure settings. Fluorescence signals were adjusted using Axiovision (Rel. 4.8) software to reduce background fluorescence while maintaining detection sensitivity; representative images are shown in the figures. All imaging experiments were performed in at least three independent replicates, and 30–50 cells were analysed per stage to determine PbARK1-GFP localisation. For additional details, refer to the figure legends.

### P. berghei structured illumination microscopy

Formaldehyde-fixed (4%) gametocytes were stained with Hoechst dye, and 2 μl of the cell suspension was placed on a microscope slide and covered with a 50 × 34 mm coverslip to form a thin, immobilised cell monolayer. Imaging was performed using structured illumination microscopy (SIM) on a Zeiss Elyra PS.1 microscope with either a Zeiss Plan-Apochromat 63×/1.4 oil immersion or Zeiss C-Apochromat 63×/1.2 W Korr M27 water immersion objective, as previously described^43^.

#### Ultrastructure expansion microscopy

U-ExM was performed largely following previously published protocols^21,44–46^. In brief, to prepare U-ExM samples of *P. falciparum* schizonts, 12mm coverslips were placed in a 24-well plate and coated with poly-D-lysine (Gibco, A3890401, 0.1 mg/mL) for 1 h at 37°C, followed by three washes with ultrapure water. *P. falciparum* schizonts were seeded onto the coverslips and incubated for 30 min at 37°C. Unattached cells were removed gently, and the remaining cells were fixed using 4% (v/v) formaldehyde in PBS for 15 min at 37°C. After fixation, coverslips were rinsed with prewarmed PBS and incubated for 12h at 37°C in 1.4% (v/v) formaldehyde and 2% (v/v) acrylamide in PBS. A monomer solution consisting of 19% (w/w) sodium acrylate (Sigma-Aldrich, 408220), 10% (v/v) acrylamide (Sigma-Aldrich, A3553), and 2% (v/v) N,N′-methylenebisacrylamide (Sigma-Aldrich, M7279) in PBS was prepared 24 h in advance and stored at − 20°C. For polymerization, 5 μL of 10% (v/v) TEMED (Sigma-Aldrich, T9281) and 5 μL of 10% (w/v) APS (Sigma-Aldrich, A3678) were mixed with 90 μL of the thawed monomer solution. The gel mix (35 μL) was pipetted onto parafilm squares pre-cooled at − 20°C and the coverslip was placed over the drop. Gels were allowed to polymerize for 1 h at 37°C. Polymerized gels were transferred to denaturation buffer (200 mM SDS, 200 mM NaCl, 50 mM Tris, pH 9) and shaken for 15 min at room temperature, then incubated at 95°C for 90 min. Denatured gels were expanded by sequential incubations in water (30 min each, repeated thrice) with gentle shaking. Expanded gels were equilibrated in PBS for 15 min and blocked in 3% BSA/PBS for 30 min at room temperature. Incubation with primary antibody (details in Supplementary Table 4) was carried out for 12h at room temperature on a shaker, followed by three 10 min washes with PBST. Gels were then incubated with secondary antibodies, 405 NHS Ester (Thermo Fisher, A30000) and SYTOX/Hoechst in PBS for 2.5 h in the dark with gentle shaking followed by three PBST washes and final equilibration in MilliQ water (30 min, repeated thrice).

For imaging, μ-slide 2-well ibiTreat chambers (ibidi, 80286) were coated with poly-D-lysine (0.5 mL, 1 h at 37°C), rinsed with MilliQ water, and used to mount the gels cell side down. Imaging was performed using a Zeiss LSM980 confocal microscope with a 63X oil immersion objective (NA 1.4). Z-stacks (0.5 μm intervals) were captured and processed using ZEN Blue (versions 3.1 or 3.9). Deconvolution was performed using Zen Deconvolution tool kit. U-ExM images are displayed as maximum intensity projections of full z-slices unless indicated otherwise.

To prepare U-ExM samples of *P. berghei* male gametocytes, purified and 8 min- and 15 min-activated gametocytes were fixed in 4% formaldehyde in PHEM buffer (60 mM PIPES, 25 mM HEPES, 10 mM EGTA, 2 mM MgCl_2_, pH6.9) at room temperature for 15 min. Fixed samples were attached to 10 mm round Poly-D-Lysine coated coverslips for 15 min. Coverslips were incubated overnight at 4°C in 1.4% formaldehyde (FA)/ 2% acrylamide (AA). Gelation was performed in ammonium persulfate (APS)/TEMED (10% each)/monomer solution (23% sodium acrylate; 10% AA; 0,1% BIS-AA in PBS) on ice for 5 min and at 37°C for 30 min. Gels were denatured for 15 min at 37°C and for 90 min at 95°C in denaturation buffer (200 mM SDS, 200 mM NaCl, 50 mM Tris, pH 9.0, in water). After denaturation, gels were incubated in distilled water overnight for complete expansion. The following day, circular gel pieces with a diameter of ~ 13 mm were excised, and the gels were washed in PBS three times for 15 min to remove excess water. The gels were then incubated in blocking buffer (3% BSA in PBS) at room temperature for 30 min, incubated with mouse monoclonal anti-a-tubulin antibody (T9026, Sigma-Aldrich, 1:500 dilution) and/or anti-GFP antibody (A11122, Invitrogen, 1:500 dilution) in blocking buffer (1:500 dilution) at 4°C overnight and washed three times for 15 min in wash buffer (0.5% v/v TWEEN-20 in PBS). The gels were incubated with 8 μg/ml Atto 594 NHS ester (08741, Merck), 10 μg/ml Hoechst 33342 (H1399, Invitrogen), Alexa Fluor 488 goat anti-mouse IgG (A11001, Invitrogen, 1:500 dilution) and Alexa Fluor 568 goat anti-mouse IgG (A11004, Invitrogen, 1:500 dilution) in PBS at 37°C for 3 hours followed by three washes of 15 min each in wash buffer (blocking and all antibody incubation steps were performed with gentle shaking). The gels were then washed three times for 15 min with wash buffer and expanded overnight in ultrapure water. The expanded gel was placed in a 35 mm glass bottom dish (MATTEK) with the 14 mm glass coated with Poly-D-Lysine and mounted with an 18 × 18 mm coverslip to prevent the gel from sliding and to avoid drifting while imaging. High-resolution confocal microscopy images were acquired using a Zeiss Celldiscoverer 7 with Airyscan using a Plan-Apochromat 50 ×/1.2NA Water objective, with 405, 488 and 561 nm lasers. Confocal z-stacks were acquired using line scanning and the following settings: 55 × 55 nm pixel size, 170 nm z-step, 2.91 μs/pixel dwell time, 850 gain and 3.5% (405 nm), 4.5% (488 nm) and 5.0% (561 nm) laser powers. The z-stack images were processed and analysed using Fiji (Version 1.54f)^47^.

#### Calculation of the expansion factor for the U-ExM of male gametocytes

The expansion factor for the U-ExM of male gametocytes was calculated by comparing axoneme diameters from two types of images (U-ExM and TEM). The diameters were measured using Fiji (Version 1.54f)^47^. The average diameter of the axoneme in NHS-stained U-ExM images of male gametocytes activated for 8 min was 933.83 nm (n = 12), while the average diameter in TEM images of male gametocytes activated for 8 min was 188.50 nm (n = 8). We used the calculated expansion factor of 4.954 for the analysis.

#### Electron microscopy of male gametocytes

*P. berghei* gametocytes activated for 8 min and 15 min were fixed in 3% glutaraldehyde in 0.1 M cacodylate buffer and processed for electron microscopy^48^. Briefly, samples were post-fixed in 1% aqueous osmium tetroxide, treated en bloc with 2% aqueous uranyl acetate, dehydrated in graded ethanol series and embedded in Spurr’s epoxy resin. Thin sections were stained with lead citrate prior to examination in a Tecnai G2 12 BioTwin (FEI UK, UK) or a Jeol JEM-1400Flash (JEOL, Japan) electron microscope.

#### Liver stage parasite imaging

For *P. berghei* liver-stage imaging, 100,000 Hepatocelluar carcinoma cells (HepG2) were seeded onto glass coverslips in 48 well plate. Salivary glands from *Anopheles stephensi* mosquitoes infected with PbARK1-GFP parasites were dissected and homogenized using a pestle to release sporozoites. The sporozoites were gently pipetted onto the HepG2 cell monolayer and incubated at 37°C with 5% CO_2_ in complete minimum Eagle’s medium The culture medium was replaced 2 hours post-infection and subsequently changed daily. For live-cell imaging, Hoechst 33342 (Molecular Probes) was added to a final concentration of 1 μg/ml to stain host and parasite nuclei. Infected cells were imaged at, 48-, and 64 -hours post-infection using a Zeiss Axio Imager M2 microscope equipped with a 63× oil immersion objective and an AxioCam ICc1 digital camera (Carl Zeiss) using autoexposure settings. and Leica Application Suite X software. Each experiment was performed in triplicate, and 20–30 infected cells were analysed per time point to assess PbARK1-GFP localisation.

#### Immunoblotting

*P. falciparum* - Immunoblotting was performed as described previously^49^ using primary antibodies (Supplementary Table 4) and HRP-conjugated secondary antibodies and protein bands were visualized using either SuperSignal^™^ West Pico or Femto chemiluminescent substrates (Pierce, USA) using X-ray films.

*P. berghei* - Purified gametocytes were lysed in buffer containing 10 mM Tris-HCl (pH 7.5), 150 mM NaCl, 0.5 mM EDTA, and 1% NP-40. After lysis, Laemmli buffer was added, and samples were boiled for 10 min at 95°C, followed by centrifugation at 13,000 × g for 5 min. The supernatants were separated on a 4–12% SDS-polyacrylamide gel and transferred onto a nitrocellulose membrane (Amersham Biosciences). Immunoblotting was performed using the Western Breeze Chemiluminescence Anti-Rabbit Kit (Invitrogen), following the manufacturer’s protocol. An anti-GFP polyclonal antibody (Invitrogen) was used at a 1:1,250 dilution to detect PbARK1-GFP.

#### Quantitative Real-Time PCR (qRT-PCR) analyses

*P. falciparum* - Total RNA was extracted using TRIzol reagent (G Biosciences) followed by purification with the Qiagen RNeasy Mini Kit (Cat. No. 74104). cDNA synthesis was performed using RevertAid H Minus Reverse Transcriptase (Thermo scientific, EP0451) and 1 μg of DNase-treated RNA as the template. Quantitative PCR was conducted using the CFX96 Real-Time PCR Detection System (Bio-Rad). Reactions were normalized using 18S rRNA as the internal control. Relative gene expression levels were calculated using the 2^−ΔΔCt^ method. Primer sequences used for the qRT-PCR are listed in Supplementary Table 3.

*P. berghei* - Total RNA was extracted from gametocytes using the RNA Purification Kit (Stratagene), and cDNA was synthesised using the RNA-to-cDNA Kit (Applied Biosystems). qPCR was performed with 80 ng of RNA using SYBR Green Fast Master Mix (Applied Biosystems) on an Applied Biosystems 7500 Fast system. Cycling conditions were: 95°C for 20 s, followed by 40 cycles of 95°C for 3 s and 60°C for 30 s. Primers were designed using Primer3 (https://primer3.ut.ee/). Each gene was tested in three biological and technical replicates. *hsp70* (PBANKA_081890) and *arginyl-tRNA synthetase* (PBANKA_143420) were used as reference genes. Primer sequences are listed in Supplementary Table 3.

#### Transcriptome analysis using RNA-seq

Total RNA was extracted from activated gametocytes and schizonts of *P. berghei* WT-GFP and *ark1PTD* parasites (three biological replicates each) using the RNeasy Kit (Qiagen), vacuum-concentrated, and transported in RNA-stable tubes (Biomatrica). Strand-specific mRNA libraries were prepared with the TruSeq Stranded mRNA Sample Prep Kit (Illumina) and sequenced on an Illumina HiSeq 4000 platform (paired-end, 150 bp reads). Read quality was assessed with FASTQC, and trimming of low-quality reads and adapter sequences was done using Trimmomatic^50^. Reads were mapped to the *P. berghei* ANKA genome (PlasmoDB release 40) using HISAT2 v2.1.0^51^, and gene counts were generated with FeatureCounts^52^. Low-expression genes (CPM < 1) were excluded. Data were normalized using the TMM method (EdgeR^53^), transformed with voom (limma^54^), and analysed for differential expression using DESeq2^55^. Genes with fold-change > 2 and FDR < 0.05 were considered differentially expressed.

#### Immunoprecipitation and mass spectrometry

*P. berghei* purified schizonts and gametocytes (activated for 1.5 to 2 min) were crosslinked using formaldehyde (10-min incubation with 1% formaldehyde, followed by 5-min incubation in 0.125M glycine solution and 3 washes with PBS (pH 7.5). Immunoprecipitation was performed using crosslinked protein and a GFP-Trap_A Kit (Chromotek) following the manufacturer’s instructions. Proteins bound to the GFP-Trap_A beads were digested using trypsin and the peptides were analysed by LC-MS/MS and subsequently visualised using both Principal Component Analysis (PCA) and enrichment visualised using a volcano plot^43,56^.

#### Evolutionary bioinformatics

Our predicted proteome set was based on a version previously used (see Supplementary Table 5). Phyletic profiles for ARK1–3 and INCENP were partially derived from previous studies^10,57^. Sequence searches for missing orthologues were performed with the HHPred webserver from MPI-toolkit^58,59^ and the hmmer package^58^, as previously described^60^. For the larger gene families like Aurora kinases, we used IQ-tree-based maximum-likelihood phylogenetics (standard setting - model finding, 1000 bootstraps^61^) to check the validity of bidirectional-best-blast (BBH) hits amongst our genome sets on an ad hoc basis. Off note, we could not reliably generate a single phylogenetic tree harbouring all three ARKs within the Aurora kinase orthologous group (OG), suggesting that ARK2 and ARK3 are either extremely divergent or not Aurora kinases. Similarly, for the INCENP gene family, multiple alignments were generally too divergent and short to infer any consistent phylograms. All sequences used for this study can be found in Supplementary Table 5.

#### AlphaFold3 modelling

Co-folds of 3D protein structures for ARK1 and the extended IN-box of either INCENP1 or 2 were modelled using the AlphaFold3 webserver (https://alphafoldserver.com/) with standard settings (seed set to 100)^62^.

## Supplementary Material

Supplementary Files

This is a list of supplementary files associated with this preprint. Click to download.

- SupplementaryFileslegends.docx
- ExtendedDataFigs.pdf
- SupplementaryVideo1.mp4
- SupplementaryVideo2.mp4
- SupplementaryVideo3.mp4
- SupplementaryVideo4.mp4
- SupplementaryVideo5.mp4
- SupplementaryVideo6.mp4
- SupplementaryVideo7.mp4
- SupplementaryVideo8.mp4
- SupplementaryTable1.xlsx
- SupplementaryTable2.xlsx
- SupplementaryTable3.xlsx
- SupplementaryTable4.xlsx
- SupplementaryTable5.xlsx

## Data Availability

The RNA-seq data generated in this study have been deposited in the NCBI Sequence Read Archive with accession number: PRJNA1309997.
